# Assessment of prescription pattern and prescription error in outpatient Department at Tertiary Care District Hospital, Central Nepal

**DOI:** 10.1186/s40545-019-0177-y

**Published:** 2019-07-10

**Authors:** Rajeev Shrestha, Srijana Prajapati

**Affiliations:** Department of Pharmacy, Bharatpur District Hospital, kuber marga, Bhagawaoti Tol, Bharatpur-04, Chitwan, Nepal

**Keywords:** Hospital pharmacy, Prescription error, Prescription pattern, WHO indicators

## Abstract

**Background:**

The rational use of medicine improves patient’s quality of life. Excessive and inappropriate prescriptions result in severe consequences. The study of drug use patterns and prescription errors is necessary to promote rational drug use in developing countries. The aim of the study was to evaluate prescription practice and help to the quality use of medicine.

**Methods:**

A retrospective, cross-sectional and quantitative study was conducted at Bharatpur District Hospital in central Nepal. The outpatient prescriptions retained at the pharmacy from November to December 2017 were used to evaluate prescription patterns and errors. The total of 770 prescriptions were reviewed. The stratified random sampling method was used.

**Result:**

The total of 2448 drugs were prescribed in 770 prescriptions or patients. The average number of drugs per encounter was 3.2. The percentage of encounter with antibiotic and injection was 37.9% (*n* = 292) and 0.7% (*n* = 5), respectively. The percentage of drugs prescribed by generic and from an essential medicine list of Nepal was 2.9% (*n* = 72) and 21.3% (*n* = 521), respectively. The most common 32.5% of prescriptions contain three drugs and 24.7% of prescriptions contain four drugs. The average prescription errors per prescription were 3.4. Among total errors, omission errors related to prescriber were 1.5 (*n* = 1135), omission errors related to the drug were 1.5 (*n* = 1189) and commission errors were 0.3 (*n* = 269). The total of 249 drug interactions were found in 19.1% (*n* = 147) prescriptions. The common prescription errors were due to failure to mention prescriber name 87.5% (*n* = 674), failure to mention prescriber signature 19.2% (*n* = 148) and failure to mention diagnosis 39.2% (*n* = 302).

**Conclusion:**

The study shows low compliance with WHO prescribing indicators and high prescription errors. The prescribing practices were not confirmed to the standard recommended by WHO. Prescribing from the Essential Drug List (EDL), low rates of generic prescribing, high antibiotic prescribing and polypharmacy were a major problem. The study found major errors in prescriber and prescribed medicine details. Remarkable drug interactions were seen in prescribed medicines. The study recommended necessary practices and policy formulation and implementation by DTC and regulatory bodies to promote the rational use of medicine.

## Background

The goal of drug therapy is to improve a patient’s quality of life. Medicine plays a vital role in drug therapy. The drug should be used in the right way knowing what medicine is right for a patient at the right dose for adequate periods as per clinical need [1]. DTC can significantly improve drug use and reduce costs in health care facilities. Inappropriate use of medicine wastes resources and diminishes the quality of patient’s care [2]. Essential drugs are safe, efficacious and affordable. The access and rational use of essential medicine is a better way to improve health status [3].

The prescription is a legal document comprising instructions for medication by a licensed medical practitioner to the pharmacist [4]. The prescription writing guidance is given in BNF, WHO practical manual on prescribing and Medical Council ethical codes of Nepal [5–7]. A previous study showed that the majority of physicians don’t adhere to guidelines made by the regulatory body. The correct prescription has a tremendous influence on drug therapy as well as patient’s health [8, 9]. 

The study on prescribing medicine is a standard process defined by WHO, which measures health care provider’s performance related to the appropriate use of drugs. The study of drug use patterns by the use of WHO indicator is necessary to promote rational drug use in developing countries. WHO indicators are globally accepted methods, which have been used in over 30 developing countries. Core prescribing indicators aimed at measuring the degree of poly-pharmacy, a tendency to prescribe in generics, levels of antibiotic use and injection and the degree of drugs prescribed from the essential drug list [3, 10] . The previous study from a teaching hospital in Nepal reported higher average drug per prescription, lower generic drug prescriptions, higher antibiotic prescriptions and less prescribing from WHO EDL [11]. 

The prescription errors are mainly of two types, errors of omission and errors of commission. Errors of omission mean prescription missing essential information, while errors of commission mean wrongly written information in the prescription [9]. DDIs are defined as a significant reduction of potency or efficacy of drugs by combining two or more drugs to patients. Drug interaction results in toxic effects or therapeutic failure. DDIs cover 6–30% of all adverse drug events [12]. 

The prescribing error is an avoidable medication error common in hospitals worldwide. The study revealed errors in 1.5% of medications ordered in hospital stays in the UK and up to 6.2% in the USA [13]. The prescription error was found to account for 70% of medication errors [14]. The study showed one-third of adverse drug reaction(ADR), which occurred due to medication errors (MEs) in Saudi Arab [15]. The National Coordinating Council for Medication Error reporting and prevention reported 15% of the medication errors are because of handwriting problem, abbreviations problem and incomplete medication orders. [4] The study in eastern Nepal reported at least one or more errors per prescription. [16] A study of geriatric patients at a teaching hospital in Nepal reported a higher number of potential prescription errors. They found an average of 0.37 drug interactions per patient and at least one potentially inappropriate medication prescribed to 53% of patients. [17] These studies emphasised the need for periodic monitoring and evaluation to minimise errors.

Excessive and inappropriate prescribing results in severe consequences such as wastage of the public economy, increased risk of toxicity, increased adverse drug reaction, increased antimicrobial resistance and decreased faith in the medical profession [18]. Poor communication between patients and prescribers, self-medication and unethical medicine promotion has been reported to increase irrational prescribing [12]. The pharmacists play a vital role in the detection and prevention of potential prescription errors. A computerised prescription order entry system along with a manual review of medication and pharmacist participation in physician rounds can improve medication safety [19].

The study of prescription patterns and errors has not been done at Bharatpur District Hospital before. Therefore, the present study will help to understand the prescribing practices and errors, which will lead to developing a proper health care policy; which will, in turn, improve the quality of the use of medicine and healthcare facilities.

## Methodology

### Study setting, design and period

Bharatpur district hospital is a tertiary care governmental hospital situated in the central Terai region of Nepal. It is the government referral hospital for the surrounding district of Province number 3. The average outpatient visits per day range from 600 to 900. A retrospective, cross-sectional and quantitative survey designed to describe the current prescribing patterns and prescribing errors at Bharatpur District Hospital, Nepal. As the retrieving of old manual retrospective data was difficult, the month of November and December 2017 was chosen randomly for sample selection.

### Data collection and analysis

The trained pharmacy personnel (1 assistant pharmacist and 1 pharmacist working in same hospital) collected data on prescribing indicators and errors retrospectively. Information on prescriptions was recorded separately for individual patients. Prescriptions from outpatient departments were included in the study. The prescription of discharged patients and admitted patients were excluded. The sample sizes of 770 were calculated with 99% confidence and 4% margin of error from total 2960 prescriptions using an online survey-size calculator [20]. The sample was selected using stratified random sampling. Firstly, the total prescriptions were divided according to the department and sample from each department was selected randomly by dividing the sample number for each department based on the prescription number of each department. The tools were pre-tested for accuracy before the data collection. All the necessary data for the study were manually analyzed first and then using Microsoft Excel 2010. The study results are expressed in number, average, and percentage form on the table below.

### Prescribing indicators

The WHO prescribing indicators are used in this study. Prescribing indicators include the average number of drugs per encounter, the percentage of drugs prescribed by generic name, the percentage of encounters with antibiotics prescribed, the percentage of encounters with injection prescribed and the percentage of drugs prescribed from essential drug list of Nepal 2016 [10, 21].

### Prescription errors

The prescription error parameters were prepared by studying WHO practical manual on guide to good prescribing and previous studies [6, 9, 16, 22, 23]. The prescription errors are classified as omission errors related to prescriber (including patient name, age, prescriber name, prescriber signature, patient visited department and diagnosis), omission errors related to drugs (including route, dose, frequency, dosage form and quantity to supply) and commission errors (including wrong strength, wrong drug name not spelling, drug dosage form and drug-drug interaction). Drug-drug interactions are classified into serious, monitor closely and minor using drug interaction checker provide by Medscape [24]. The interaction of fixed dose combination (FDC) was excluded. They were found only four.

### Operational definitions

According to the WHO manual in this study, antibiotic refers to the penicillin, cephalosporin, miscellaneous antibiotics, and dermatological anti-infective with exclusion to antihelminthic, antifungal and antidiarrhoeal except metronidazole. The metronidazole is included although it is commonly used as antiprotozoal but also used as antibacterial.

### Ethical consideration

Ethical approval was obtained from the Institution Review Board of Bharatpur District Hospital.

## Result

A total of 2448 drugs prescribed in 770 prescriptions were assessed retrospectively. The average number of drugs per prescription and percentage of encounters with antibiotics were greater than the WHO standard. However, the percentage of encounter with injection, drugs prescribed by generic, and drugs prescribed from essential drug list were lower than the WHO standard (Table 1) [25].

**Table 1:** Prescribing indicators (*n* = 770)

Prescribing Indicators assessed	Total drugs/encounters	Average/Percentage	WHO standard derived or ideal (%)
Average number of drugs per encounter	2448	3.2	1.6–1.8
Percentage of encounter with antibiotics	292	37.9%	20–26.8%
Percentage of encounter with injection	5	0.7%	13.4–24.1%
Percentage of drugs prescribed by generic	72	2.9%	100%
Percentage of drugs from essential drug list	521	21.3%	100%

The number of drugs prescribed per prescription ranges from 0 to 9. The maximum of nine medicines (0.1%) were prescribed in one prescription. Only a single prescription was found to be the absence of a drug. The 37.9% of total prescription contained antibiotics. The maximum three antibiotics were prescribed per prescription, among which one antibiotic per prescription 31.8% (*n* = 245) was highest. (Table 2).

**Table 2:** Degree of Medicine prescribed (*n* = 770)

Number of Medicine per prescription	Frequency (Percentage)	Number of Antibiotics per prescription	Frequency (Percentage)
0	1 (0.1%)	0	478 (62.1%)
1	57 (7.4%)	1	245 (31.8%)
2	165 (21.4%)	2	44 (5.7%)
3	250 (32.5%)	3	3 (0.4%)
4	211 (27.4%)		
5 and above	86 (11.2%)		
Total	770 (100%)	Total	770 (100%)

The total prescribed medicine items were 325 of which 28 were antibiotics. A total of 2448 medicines were prescribed in 770 prescriptions, in which a total of 13.9% (*n* = 339) were antibiotics. Among the frequently prescribed medicines, two were gastrointestinal antiulcer agents, three were anti-inflammatory analgesic, two were anti-allergic anti-histamines and three were antibiotics. Among the frequently prescribed antibiotics, cephalosporin antibiotics were three, while macrolide and penicillin antibiotics were two and the remaining others were one (Table 3).

**Table 3:** Frequently prescribed medicines (*n* = 2448) and antibiotics (*n* = 339)

S.N.	Frequently prescribed medicine	Frequency (Percentage)	S.N.	Frequently prescribed Antibiotic	Frequency (Percentage)
1	Pantoprazole	139 (5.7%)	1	Azithromycin	68 (20.1%)
2	Rabeprazole	127 (5.2%)	2	Amoxicillin + Clavulanic acid	65 (19.2%)
3	Ibuprofen + Acetaminophen	102 (4.2%)	3	Cefixime	48 (14.2%)
4	Levocetirizine	82 (3.3%)	4	Amoxycilin	25 (7.4%)
5	Fexofenadine	79 (3.2%)	5	Mupirocin	17 (5%)
6	Azithromycin	68 (2.8%)	6	Levofloxacin	15 (4.4%)
7	Amoxicillin + Clavulanic acid	65 (2.7%)	7	Cefadroxil	11 (3.2%)
8	Aceclofenac	56 (2.3%)	8	Clarithromycin	10 (3%)
9	Cefixime	48 (2.0%)	9	Cefpodoxime	8 (2.4%)
10	Diclofenac	48 (2.0%)	10	Doxycycline	8 (2.4%)
			11	Metronidazole	8 (2.4%)

A total of 2593 prescription errors were noted in 770 prescriptions, which means an average of 3.4 errors per prescription. The most common error in prescribing was errors of omission related to drug 1189 that is 1.5 average errors per prescription. The most common error of omission related to prescriber was failure to mention prescriber’s name which was 87.5% (*n* = 674) in total prescription. The most common error of omission related to the drug was due to a failure to mention dose which was 32.6% (*n* = 798) in total drug prescribed. The most common error of commission was due to drug-drug interaction which was 10.2% (*n* = 249) in total drug prescribed (Table 4).

**Table 4:** Prescription error

Types of Errors	Number of errors (Percentage)	Average error per prescription
Errors of omission related to prescriber (*n* = 770)		
Patient name not mentioned	0 (0%)	0
Age not mentioned	5 (0.6%)	0
Prescription date not mentioned	6 (0.8%)	0
Prescriber name not mentioned	674 (87.5%)	0.9
Prescriber signature not mentioned	148 (19.2%)	0.2
Department not mentioned	0 (0%)	0
Diagnosis not mentioned	302 (39.2%)	0.4
Total Error	1135	1.5
Errors of omission related to drugs per total medicine dispensed (*n* = 2448) and per prescription (*n* = 770)		
Dose not mentioned	798 (32.6%)	1.0
Frequency not mentioned	27 (1.1%)	0
Dosage form not mentioned	110 (4.5%)	0.1
Quantity to supply not mentioned	254 (10.4%)	0.3
Total Error	1189	1.5
Errors of commission per total medicine dispensed (*n* = 2448) and per prescription (*n* = 770)		
Wrong strength	11 (0.5%)	0
Wrong drug name (not spelling)	4 (0.2%)	0
Wrong Dosage form	5 (0.2%)	0
Drug-drug Interaction	249 (10.2%)	0.3
Total Error	269	0.3

Out of 2448 medicines prescribed in 770 prescriptions, 249 drug interactions were found in 19.1% (*n* = 147) prescriptions. Among them, monitor closely type of interaction was mostly occurred which was 62.7% (*n* = 156). The most common serious interaction was between digoxin and metoprolol (0.8%) while the most common monitor closely interaction was between Ibuprofen and salbutamol (albuterol) (2.4%) and the most common minor interaction was between rabeprazole and methylcobalamin (4%). There was a maximum of seventeen drug interactions in each prescription (Table 5).

**Table 5:** DDIs according to Medscape (*n* = 249)

Intensity of Interaction	Number (%)
Serious	11 (4.4%)
Moderate	156 (62.7%)
Minor	82 (32.9%)

## Discussion

No prescribing indicators assessed were matched with the value given by the WHO. The average number of drugs per encounter was higher (3.2) compared to the standard value 1.6–1.8 and the previous study of the Nepal 2.1 [25, 26]. The average drug per prescription was found low in a teaching hospital in Western Nepal 2.5, tertiary care hospital of India 3.03 and Nigeria tertiary hospital 3.04 [11, 18, 27]. The study showed that most of prescription that is 32.5% had three drugs and 27.5% had four drugs. In contrast, the government hospital in Ethiopia reported 36.4% of prescriptions had two drugs and 30.5% of prescriptions had one drug [28]. Similarly, PHC of India also showed lesser value i.e. 37.3% of prescriptions had three drugs and 27.8% of prescriptions had two drugs per prescription [29]. The study showed a maximum of nine drugs per prescription, which is quite high. The polypharmacy might be due to lack of therapeutic knowledge to the prescriber, prescriber carelessness toward the possible adverse effects of medicine, lack of clinical practice guidelines or lack of therapeutically correct medicine. Low drug prescribing can reduce the chances of drug interaction, unwanted adverse effects, non-compliance by patient, bacterial resistance and financial burden to the patient. Additionally, an increase in medication reported the risk of medication errors [30]. Therefore, appropriate regulation monitoring of drug therapy and evidence-based clinical guidelines are essential for avoiding an unnecessary burden to patient economy and health through a minimum number of therapeutically required drug prescribing.

The overall antibiotic prescribing per encounter 37.9% was higher than the WHO standard and lower than the previous study 43% [25, 26]. The lower value was reported in tertiary care hospital of Western Nepal 28.30%, Chinese county hospital 29.9% and tertiary care hospital of Nigeria 34.4% [11, 27, 31]. The more than single antibiotic was found to be prescribed in 6.1%, which is higher than the western UP of India 4.64% [32]. The hospital has a practice of prescribing antibiotics without sensitivity study to outpatients and did not have the hospital’s own policy on antibiotic utilization. This practice may have promoted higher use of antibiotics, which ultimately develop bacterial resistance and increases the necessity to use expensive antibiotics. A study in a tertiary care hospital in Mangalore reported 19.44% that the antibiotics prescribed were lower than the standard range. The antibiotic policy was attributed to the low rate of antibiotic prescription [18]. The evidence-based appropriate antibiotic policy is a current need for health care facilities to reduce inappropriate antibiotic use and consequent effects.

The injection prescribing was lower 0.7% than the WHO standard value, tertiary care hospital of India 8.33%, of western Nepal 3.1% and previous study 5% [11, 18, 25, 26]. The probable reason might be that non-parenteral medication use is easy, cost-effective and convenient in the busy outpatient department while the injection requires trained personnel. The lesser use of parenteral preparation decreases the chances of infection through parenteral route and reduces cost too as the parenteral preparations are more expensive than oral preparation.

The drug prescribing in the generic name was very poor 2.9% while the WHO standard was 100 and 44% in an earlier study [25, 26]. The similar study found generic prescribing 13% in western Nepal, 96.12% in Chinese county hospitals and 100% in the public hospital of Ethiopia [11, 31, 33]. However, the hospital pharmacy guidelines, medical council ethical code and WHO manual on good prescribing directs to prescribe in the generic name except if there is a particular reason to prescribe a special brand [6, 7, 34]. The study among medical students in Nepal reported that 82% of them would be influenced by a brand name due to the pharmaceutical company’s advertisement. Similarly, a study of Pakistan revealed that the majority of medical sales representatives demand prescribers to prescribe their brands and prescribers want gifts, samples, incentives and inducements from them. Pharmaceutical representative influences the prescribing pattern significantly and they are biased toward brand name medicine, which creates a negative attitude toward generic medicine. The regulatory body must take initiation to provide authentic evidence-based information about generic and branded medicine to medical prescribers [35, 36]. Generic prescribing and promotion is desired in developing countries like Nepal as it substantially reduces medicine cost and patients can easily get it as they are not compelled to look for specific medicine with a brand name. The brand prescribing has created an environment to select medicine based on prescriber prescribed brand rather than assessing quality parameters by pharmacist and DTC in hospital pharmacy. The drugs are to be assessed and selected based on their efficacy, effectiveness, safety, quality, and cost of use. The DTC is responsible for hospital setting to prepare and implement an appropriate policy on quality medicine selection, use, and monitoring [2].

Prescribing medicine from the essential drug list was very little 21.3% while it was 32.8% in western Nepal, 96.6% at Hawassa University Hospital of Ethiopia and 94.0% in tertiary care hospital of Nigeria [3, 11, 27]. The reason might be the lack of knowledge about essential medicine or promotion of newer molecule by the pharmaceutical company. However, essential medicines are cost-effective, qualitative and safe. Therefore it’s awareness and promotion by prescriber can reduce drug interaction, adverse drug reaction, maximize affordability and ultimately enhances patient financial and therapeutic benefit. The essential medicine concept was recognized as a highly rational and sensible strategy to provide evidence-based modern, cost-effective health care. The implementation of EM policy has been shown to improve the quality of medicine use, particularly in low-income countries [28, 37].

The most commonly used medicine was pantoprazole followed by rabeprazole and ibuprofen+acetaminophen. Looking categorically, the top ten most commonly used medicines were gastrointestinal antiulcer agents, analgesic anti-inflammatory agents, anti-allergic anti-histamine and antibiotics. Similarly, the most common categories of medicine reported by the teaching hospital of western UP, India were NSAIDs + serratiopeptidase 20.67%, antibiotics 17.48% and antihistamine 15.38% [32]. Likewise, the governmental hospital of Ethiopia also showed antimicrobial 39.02%, analgesics 29.67% and gastrointestinal agents 10.64% as highly prescribed categories of medicine [28]. The reason for the higher use of analgesics, antibiotics, and antiulcer agent might be due to higher infections disease, gastroenteritis, and pain or inflammation patients. The reason for the higher prescription could be due to pressure of patients seeking rapid symptomatic relief of symptoms, overestimation of the severity of illness or sometimes competition between clinicians exacerbates irrational prescribing [29]. The analgesic anti-inflammatory agents can cause a serious effect on the gastrointestinal tract, cardiac and renal system if taken inappropriately. The study reported nearly 26,000 annual deaths in America due to inadvertent misuse of NSAIDs [38]. The inappropriate long-term use of PPIs can increase adverse effects rather than benefits such as gastrointestinal bleeding, enteric and pneumonia infections, nutritional deficiencies, rebound hypersecretion etc. The previous study suggested histamine receptor type-2 blockers and lifestyle modifications are effective and sufficient in many conditions than PPIs, which reduces the economic burden and adverse effects on patients [39, 40]. Therefore, the patient’s medication need must be assessed properly to prevent its unwanted adverse effects. Otherwise, medicine can cause side effects more than healing patient illness.

Regarding the patient’s details on prescription, diagnosis 39.2% were highly missed components than others. The diagnosis missing was less in Bahalpur Pakistan 37.3% and Saudi Arabia 15.1% [41, 42]. The diagnosis was the responsibility of prescriber to mention in prescription, which was absent in more than half of prescription. The professional code of ethics insists on informing patients regarding their ailment and provides a clear explanation of diagnosis [7]. The determination of diagnosis is a part of rational prescribing. After that, therapeutic objectives have to specify and choose treatment of proven efficacy and safety [6]. If the diagnosis were not correct, the treatment would not be achieved. The wrong diagnosis results in economic wastage and patient health hazards. The diagnoses in prescription will help to dispense accurate drugs by the pharmacist during interpretation of prescription even if the handwriting of medicine mentioned is not clear [43]. The patient’s name, age, date and their visit to the department were present in almost all prescriptions. The computerized practices of collecting patient’s details have improved collecting this information.

The prescriber’s name and signature were missed in 87.5 and 19.2% prescriptions, respectively. A similar study conducted at the tertiary care hospital of India, tertiary care hospital of Nepal and Saudi Arabia showed prescriber’s name and signature were missed in 23.3 and 12%, 85.4 and 15.7%, and 16.7 and 18.1% respectively [16, 42, 44]. The prescriber details were missed comparatively higher in our study than above mentioned studies. Most of the prescriber handwriting was difficult to understand. However, readability got it possible because of dispenser familiarity with prescriber handwriting. The pharmacist or the dispenser is unable to confirm whether the prescriber and prescription are genuine or not if the prescriber details are not written in a clear, legible way. The absence of prescriber detail or illegible handwriting can promote patient to purchase a self-prescribed medication. The prescriber details are crucial in cases of narcotics, hormonal and antibiotic. These medicines can only be dispensed on the prescription of registered medical doctors. Therefore, the prescriber’s details must be required [45]. The absences of prescriber details make it difficult to communicate by pharmacists in confusion on medicine writing and by patients in further follow up on their medical conditions. The prescriber’s identity and signature must be written legibly and checked regularly before dispensing to avoid the misuse of medicine.

The present study showed that dose 32.6% and quantity to supply 10.4% were missed highly compared to frequency 1.1% and dosage form 4.5%. The dose, frequency, dosage form and quantity to supply were not mentioned in 18.9, 10.4, 12.1 and 59.9% of prescribed drug in teaching hospital of Nepal, respectively; and dose, frequency and dosage form were missed in 72.6, 15 and 67.3% of prescribed drug in governmental hospital of Ethiopia, respectively [16, 28]. Compared to other studies, dose related error is higher than of teaching hospital of Nepal, while all other errors are lower than other studies. The dose and dosage form are very important when the particular drug is available in various dose and dosage form. The clear details of a patient’s special age and diagnosis could help the dispenser or pharmacist to identify and confirm the medicine dose and dosage form to dispense when the dose and dosage form of medicine was not legible in prescription and communication to prescriber was not possible at a particular time. However, the auditing and managing the errors depends on the competency and qualification of dispenser. The inappropriate use of dose, frequency, and duration can lead to drug resistance, toxicological effect and treatment failure. Good prescribing practice is extremely important in minimizing errors in the dispensing of medications; physicians should adhere to guidelines for the benefit of patients [42].

The wrong strength 0.5%, wrong drug name 0.2%, wrong dosage form 0.2% were found less compare to drug-drug interaction 10.2%. The commission errors were low except for drug-drug interaction comparison to teaching hospital of Malaysia which showed wrong strength 0.7%, wrong dosage form 3.1% and drug-drug interactions 4.5% [22].Though the commission errors were lower compared to others, it can lead to serious consequences to patient rather than omission errors. Therefore, the prescription has to be written clearly and study sincerely before dispensing otherwise, identification of errors is untraceable and can result in harmful consequences. Medication errors may result in ADR, therapeutic failures and ultimately wasting resources. The DTC of hospital where the pharmacist plays a vital role has a role in monitoring and addressing medication errors [2]. A pharmacist must study the prescription before dispensing in order to avoid errors and consult the prescriber in case of confusion. A study done in Saudi Arabia showed 11–89% of medication errors were prevented by pharmacist intervention. Pharmacist in collaboration with other health care personnel was found to reduce medication errors remarkably [15].

The drug-drug interaction was a superior error, among other commission errors, and was found to be superior in another study as well [4]. Among total DDIs, the monitor closely category was 62.7% (*n* = 156) superior to other types of interaction and it was similar in another study [43]. Moderate types of interaction are less severe than major types but they can cause harm and treatment may also be required. The study showed a significant number of drug interactions, which were not found to be monitored and managed accordingly. A previous study by Iran also declared that ignoring drug interaction was a predominant commission error [9]. DDIs should be prevented as much as possible to reduce adverse drug events because these are responsible for 3–23% of the total hospital admission [43]. The possibility of drug interaction is higher with higher drug prescribing. The pharmacist should advise patients on possible interaction and its outcome if there is no other alternative medicine to avoid interactions. Similarly, physicians should be advised to use alternative medicine based on drug interaction. However, the presence of clinical pharmacists is not accessible in most hospitals. They have a role to monitor and evaluate the use of medicine to avoid drug interaction and adverse drug reactions [2]. The hospital directives also clarified the need for clinical pharmacists in the hospital, while the implementation is lacking [34]. The previous study has also emphasized the need for qualified personnel for the management of drug interaction [17]. The computerized prescribing order system along with drug interaction monitoring software has significance to trace and prevent drug interaction.

The study had limitations of having only the data of two months and the drug interactions were studied based on the medicine available in the online reference of Medscape. Not all the prescribed medicine in the prescription was found in an online reference of Medscape to their involvement in drug interaction. The WHO standard value was taken for study, which may not be exact for comparison because the number of medicines used may vary based on pharmacotherapeutic aspects of patients.

## Conclusion and recommendation

The study shows low compliance with WHO prescribing indicators and high prescription errors. Prescribing from the EDL, generic and antibiotic prescribing, and polypharmacy were a major problem. The most common prescription errors were the omission errors related to the drug. The study found major errors in prescriber and prescribed medicine detail. The remarkable drug interactions were found in prescribed medicines.

The study recommended strengthening the DTC with a particular emphasis on formulating policy and evidence-based clinical guideline focusing essential medicines, generic prescribing, appropriate antibiotic use and controlling polypharmacy. The study recommended the need for qualified clinical pharmacists and professional interaction to evaluate drug use, trace errors and manage accordingly. The regulatory body should work on formulating and evaluating hospital guideline implementation and policy regarding rigorous monitoring and improvement to promote rational drug utilization in a health care setting. The government has to work on the implementation of generic medicine awareness, essential medicine promotion and utilization for easy access to cost effective medicine.

## Abbreviations

ADR: Adverse drug reaction
BNF: British National Formulary
DDIs: Drug-drug interactions
DTC: Drug and therapeutic committee
EDL: Essential Drug List
MEs: Medication errors
NSAIDs: Non-steroidal anti-inflammatory drugs
PHC: Primary health centre
PPIs: Proton pump inhibitors
WHO: World Health Organization
